# Advent of Pulsed Field Ablation for Atrial Fibrillation: State-of-the-Art Review

**DOI:** 10.31083/RCM47497

**Published:** 2025-12-22

**Authors:** Francis J. Ha, Hui-Chen Han, Nitesh Nerlekar, Adam J. Brown, Emily Kotschet

**Affiliations:** ^1^Victorian Heart Institute and Victorian Heart Hospital, Monash University, Monash Health, Clayton, VIC 3168, Australia

**Keywords:** atrial fibrillation, catheter ablation, irreversible electroporation therapy

## Abstract

Pulsed field ablation (PFA) is a novel ablation technique for atrial fibrillation (AF). Indeed, PFA utilizes cell electroporation and exhibits selectivity for myocardial tissue, depending on the method used to deliver the pulsed electric field, potentially sparing surrounding non-cardiac structures. Recent clinical trials have demonstrated the non-inferiority of PFA compared with conventional thermal ablation for arrhythmia recurrence, including radiofrequency and cryoballoon ablation. Currently, large registry data present an acceptable safety profile. However, PFA is not without risk, and some unique, albeit infrequent complications have been recognized with this ablation modality, including stroke, coronary artery spasm, and intravascular hemolysis. Thus, given the associated safety, efficacy, and improved procedural workflow of this technique, the advent of PFA will likely lower the threshold for patient selection for AF ablation, particularly owing to the growing burden of AF in our community. This review provides an overview of the biophysics of PFA, various catheter designs, clinical trial and registry data, potential complications associated with PFA, and future directions in this promising area of AF management.

## 1. Introduction

Catheter ablation for atrial fibrillation (AF) has been performed for more than two decades and represents an important treatment milestone for improved symptom and rhythm control. Thermal ablation has been adopted worldwide since the seminal study by Haïssaguerre and colleagues [1] in 1998, which showed that the pulmonary veins (PVs) are a significant trigger of AF. Sham-controlled trial data revealed a substantial reduction in AF burden, improved quality of life, and symptoms [2]. At present, catheter ablation has a class 1A recommendation to improve symptoms in patients with symptomatic AF in whom anti-arrhythmic drugs (AADs) have failed or not been tolerated [3]. Similarly, patients with AF and heart failure with reduced ejection fraction also have a class 1A indication for catheter ablation, given superiority over rhythm control with drug therapy for all-cause death and heart failure hospitalization [3]. Thermal catheter ablation has been undertaken using either radiofrequency (heat) or cryoablation (freezing) with similar outcomes [4]. Nevertheless, these ablative techniques are limited by a lack of selectivity, as these techniques rely on thermal energy to induce cell injury and necrosis. This may lead to PV stenosis and damage to surrounding anatomical structures, including the phrenic nerve and the esophagus. Atrioesophageal fistula, while rare, remains a feared complication, and presents a mortality up to 55% and a lack of definitive preventive strategies [5, 6, 7].

The recent advent of pulsed field ablation (PFA) has changed the therapeutic landscape for catheter ablation for AF. PFA employs cell electroporation and shows greater selectivity for myocardial cells, causes less thermal injury when delivered appropriately, and, thus, mostly spares surrounding extracardiac structures. This differs substantially from radiofrequency or cryoablation, as the anti-arrhythmic effects are achieved not by thermal energy but by electroporation. PFA is also associated with reduced procedural and left atrial catheter dwell times and improved workflow, which facilitates same-day discharge. Growing clinical trial and post-approval registry data have shown that PFA is safe and effective compared to traditional thermal ablation techniques. This has led to increased uptake of PFA worldwide, with more than 500,000 cases performed globally since the first commercial case in 2021.

In this narrative review, we provide an overview of the biophysics of PFA, catheter design, appraisal of clinical trial and registry data, potential specific complications, procedural workflow, and future direction in performing PFA to treat AF (Table 1). Several other narrative reviews have been published in recent years on the use of PFA for cardiac arrhythmias [8, 9]. However, this review focuses specifically on PFA for AF, where the most evidence is available, with a predominant emphasis on clinical data and outcomes (rather than preclinical and benchside data) for clinical cardiologists, and highlights future directions in this field.

**Table 1.** **Research priorities in PFA for AF**.

Research priorities in PFA for AF	
1. Evolving catheter design	
	a. Improvement in sheath size and number of sheath exchanges
	b. Steerability within the left atrium
	c. Reducing air bubble entrainment
	d. Integration of three-dimensional electroanatomic mapping
	e. Integration of tissue-contact sensing design
2. Monitoring for complications	
	a. Ongoing long-term registry data to identify rare complications
	b. Evaluation of potential subclinical effects (e.g., coronary spasm, intravascular hemolysis)
3. Lesion optimization and appropriate ablation strategy	
	a. Lesion durability
	b. Role of additional cardiac imaging (e.g., intracardiac echocardiography)
	c. Utility of posterior wall isolation and other non-PV triggers in patients with persistent AF using PFA
	d. Efficacy and safety of CTI and mitral isthmus ablation
4. Safety and efficacy of PFA in specific patient groups	
	a. Older patients (particularly ≥75 years)
	b. Younger patients
	c. Patients with obesity
	d. Sex-specific clinical outcomes
	e. First-line catheter ablation with PFA versus medical therapy in paroxysmal AF

AF, atrial fibrillation; CTI, cavotricuspid isthmus; PFA, pulsed field ablation; PV, pulmonary vein.

## 2. Biophysics of Pulsed Field Ablation

PFA relies on cell electroporation to create ablation lesions. An electrical field is generated by the delivery of ultra-short, high-voltage impulses that invert phospholipids at the cell membrane, allowing the passage of ions and small molecules into the cell [10]. Hydrophilic pores form on the cell membrane surface through lipid oxidation, in concert with reactive oxygen species generated by the electric field, disrupting cell proteins and destabilizing the cell cytoskeleton. Other mechanisms of cell injury include Ca^2+^ influx, mitochondrial damage, and adenosine triphosphate (ATP) depletion.

PFA is sometimes incorrectly described as a non-thermal ablation technique. However, electroporation delivered at sufficiently high voltage over a certain pulse duration can still trigger tissue heating. Tissue selectivity applies up to a given threshold of voltage delivery; beyond that, other structures may still be affected. Adjustable parameters of energy delivery include voltage, pulse duration, frequency, biphasic or monophasic energy delivery, and unipolar or bipolar configuration. However, cardiomyocytes may be more susceptible to electroporation due to certain intrinsic cell characteristics, such as relatively greater ion channel expression, which can be denatured by electroporation, and a larger cell radius, which lowers the associated electric field threshold relative to smooth muscle cells (e.g., esophagus) [11]. Furthermore, PFA has a central zone of irreversible electroporation in proximity to the catheter electrode and a surrounding zone of reversible electroporation, where initial cell membrane disruption can recover over time [12]. This represents a potential limitation of the technology, as cells that are only reversibly electroporated may appear electrically silent (acute loss of local intracardiac electrograms) but may still recover over time. Indeed, PVs have been reconnected in up to 55% of patients undergoing clinically indicated repeat procedures [13]. However, this is likely overestimated compared to overall durability when including patients without symptomatic recurrences.

## 3. Pulsed Field Ablation Device Design

An ever-expanding number of PFA systems are becoming commercially available worldwide. This is a competitive landscape, with the industry driving constant design improvements. However, rather than providing an overview of all commercially available devices or devices currently in clinical trials, understanding the clinical indication for PFA use and the subsequent relevant, tailored design best suited to the purpose is perhaps more important.

In the context of AF, performing PV isolation as the standard *de novo* ablation strategy is most efficiently achieved through a degree of circumferential design. This enables efficient workflow and potentially mitigates the risk of embolic stroke. Various PFA systems deliver trains of bipolar or biphasic stimuli with an electric field strength of 1.5–2.0 kV over fixed pulse durations. Design differences include the number and shape of electrodes, the relevant electrode spacing, catheter diameter, steerability, and the incorporation of an over-the-wire design. Other factors include integration of tissue-contact sensing and three-dimensional (3D) electroanatomic mapping to guide overlapping lesion delivery, activation, and voltage mapping. While PV isolation remains the standard lesion set in paroxysmal AF, posterior wall (PW) isolation with these catheters is technically feasible.

Focal point-by-point catheters are also currently commercially available or in clinical trials. The ability to deliver focal lesions using PFA will change the treatment landscape for other ablation areas, including the mitral isthmus, cavotricuspid isthmus, and ventricle.

## 4. Clinical Trials in Pulsed Field Ablation

Growing clinical trial data generally support the use of PFA for AF ablation. Initial first-in-human, non-randomized, industry-sponsored studies (IMPULSE and PEFCAT) enrolled patients with paroxysmal, drug-resistant AF to PFA with Farapulse™ (Boston Scientific, USA) [14]. In 81 patients, 87.4% were free of arrhythmias as measured by 24-hour Holter monitoring at 12 months of follow-up. Farapulse™ has also been evaluated in 339 patients with persistent AF who underwent PV isolation and PW ablation in the ADVANTAGE AF single-arm study, achieving a 1-year freedom from arrhythmia of 63.5% [15]. Four patients developed pulmonary edema, likely related to the volume of intravenous fluids administered peri-procedurally to reduce the risk of hemolysis. The PULSED AF Pivotal trial was a non-randomized, single-arm, industry-sponsored trial that evaluated a different circumferential catheter (PulseSelect™, Medtronic, USA) [16]. In 300 patients with paroxysmal and persistent AF, freedom from a composite of acute procedural failure, arrhythmia recurrence, or AAD escalation was 66% and 55%, respectively, at 1 year. Similar findings have been reported with the Varipulse™ (Johnson and Johnson Medtech, USA) circumferential PFA system, with a 1-year freedom-from-arrhythmia rate of 78.9% in 226 patients with drug-refractory paroxysmal AF [17].

Randomized controlled trials have been conducted comparing PFA with conventional thermal ablation (Table 2, Ref. [18, 19, 20]). The ADVENT Trial was an industry-sponsored, non-inferiority trial that randomized patients with drug-refractory paroxysmal AF to either PFA with Farapulse™ versus conventional radiofrequency or cryoballoon catheter ablation [18]. Arrhythmia detection was performed using 72-hour Holter monitoring at 6 and 12 months and weekly trans-telephonic electrocardiographic recordings after a 3-month blanking period. At 1-year follow-up, there was no difference between groups for the primary composite efficacy endpoint of procedural failure, atrial tachyarrhythmia recurrence, AAD use, cardioversion, or repeat ablation. Similarly, the SINGLE SHOT CHAMPION trial was a non-inferiority trial conducted in two centers in Switzerland that randomized patients with paroxysmal AF to PFA (Farapulse™) or cryoballoon ablation (Arctic Front™, Medtronic) [19]. Importantly, all patients had a continuous cardiac monitor implanted, and the trial was investigator-initiated, although the trial received an unrestricted research grant from industry. At 1-year follow-up with a 3-month blanking period, PFA was shown to be superior to cryoballoon ablation for their primary endpoint of freedom from atrial tachyarrhythmia recurrence ≥30 seconds (63% vs. 49% at 1-year, respectively; *p* < 0.001 for non-inferiority and *p* = 0.046 for superiority).

**Table 2.** **Published randomized controlled trials comparing PFA with conventional thermal ablation for AF**.

Trial	Inclusion criteria	Intervention	Comparison	Primary efficacy endpoint	Blanking period	No. of patients	Outcomes
ADVENT (2023) [18]	≤75 years, symptomatic paroxysmal AF refractory to ≥1 AAD	PFA (Farapulse™, Boston Scientific)	Thermal ablation (RF or cryoballoon)	Freedom from composite of initial procedural failure, AT recurrence, AAD use, cardioversion, or repeat ablation	3 months	305	PFA non-inferior to thermal ablation at 1 year (73.3% vs. 71.3%)
SINGLE SHOT CHAMPION (2025) [19]	Symptomatic paroxysmal AF	PFA (Farapulse™, Boston Scientific)	Cryoballoon	AT recurrence	3 months	210	PFA non-inferior and superior (*p* = 0.046) to cryoballoon at 1 year (37.1% vs. 50.7%)
SPHERE Per-AF (2024) [20]	≤80 years, symptomatic persistent AF refractory to ≥1 AAD	Dual energy PFA–RF (Sphere-9, Medtronic)	RF	Freedom from initial procedural failure, repeat ablation, AT recurrence, escalation or initiation of class I/III AAD or cardioversion	3 months	420	Dual energy catheter non-inferior to RF at 1 year (73.8% vs. 65.8%)

AF, atrial fibrillation; AAD, anti-arrhythmic drugs; AT, atrial tachyarrhythmia (atrial tachycardia, atrial fibrillation, atrial flutter); PFA, pulsed field ablation; RF, radiofrequency.

In patients with persistent AF, the SPHERE Per-AF trial evaluated a dual-energy Sphere-9™ catheter with electroanatomic mapping (Affera™, Medtronic) compared with conventional radiofrequency (RF) ablation [20]. In this industry-sponsored, non-inferiority trial with 420 patients, composite freedom from procedural failure and repeat ablation, arrhythmia recurrence (evaluated by 24-hour Holter monitoring), drug initiation or escalation, or cardioversion after a 3-month blanking period was 73.8% and 65.8%, respectively, at 1 year.

While these are well-designed contemporary randomized trials, certain limitations should be recognized. First, these trials possessed relatively small sample sizes, given that more than 500,000 cases have now been performed worldwide using the Pentaspline catheter. Second, these studies are at least, in part, industry-sponsored. Third, a three-month blanking period is likely excessive for PFA technology, given the associated reduced proinflammatory profile compared with conventional thermal ablation. There is also an established association between early arrhythmic recurrences predicting late recurrences [21, 22, 23]. Guidelines now suggest an eight-week blanking period [24]. Fourth, there is heterogeneity in the endpoint, be it arrhythmia recurrence alone or the inclusion of repeat ablation, cardioversion, or drug initiation. Similarly, there is variability in monitoring arrhythmic recurrence, including Holter monitoring, trans-telephonic electrocardiographic monitoring, or an implantable loop recorder. Fifth, these trials have a one-year follow-up. It is recognized that most AF recurrences tend to be frontloaded, potentially arguing for a relatively short duration of follow-up for arrhythmic recurrences [25]. However, longer-term safety outcomes are still needed to assess potential late adverse sequelae, such as coronary artery stenosis [26].

## 5. Specific Potential Complications Associated With Pulsed Field Ablation

To monitor complications, large post-approval surveys and registry data on the Farapulse™ catheter have reported major adverse event rates of less than 2% and minor adverse event rates of approximately 3–4% [27, 28, 29, 30]. There have been no reported cases of atrioesophageal fistula or symptomatic PV stenosis. Symptomatic phrenic nerve injury has been transient to date. While PFA has been considered a relatively safe ablation modality due to the associated greater tissue selectivity, this technique is not without risk, as PFA is associated with certain potential, if not unique, complications.

Pericardial tamponade has an estimated risk of up to 1.14% across registry data, which places pericardial tamponade as the most common serious adverse complication, occurring more frequently than even major vascular access complications [29]. The risk of pericardial tamponade has been reported to be higher compared with thermal ablation in a meta-analysis of randomized and non-randomized studies (1% vs. 0.2%; odds ratio (OR) 2.98, 95% confidence interval (CI) 1.27–7.00) [31]. The reasons are likely multifactorial, including patient factors, catheter design, operator experience, and therapeutic anticoagulation. Meanwhile, ongoing improvement in catheter system design will likely reduce the risk of inadvertent cardiac injury.

Stroke remains one of the most feared complications related to AF ablation, and this risk persists with PFA. The risk of stroke may depend on the specific PFA catheter and operator experience. Stroke risk does not appear elevated beyond that of conventional thermal ablation, based on post-approval registry and pooled clinical trial data for the Farapulse™ catheter [32]. Nationwide German data reported a stroke rate of 0.2% with thermal ablation and <0.1% with PFA [33]. Conversely, the Varipulse™ catheter was temporarily paused in the United States due to concerns regarding stroke risk. Along the spectrum of stroke, silent cerebral emboli (SCE) have been a concern with conventional thermal ablation, and this risk persists with PFA [34]. Clinical prospective data have reported an SCE risk between 9% and 12% [16, 17]. However, a small study reported SCE in 6 of 7 patients undergoing PFA with Varipulse™, with a median of 13 lesions, ≥10 mm lesion size in three patients, and many lesions being multi-territory [35]. This was higher than that reported with PulseSelect, which reported SCE in 2 out of 9 patients (*p* = 0.04). The mechanism is potentially multifactorial, relating to air entrainment and gas bubble formation during lesion application. Additionally, given the faster procedural workflow for PFA, the peri-procedural activated clotting time may not peak sufficiently post-heparin administration to enable therapeutic anticoagulation for a substantial portion of the procedure. The long-term consequences of SCE on neurocognitive function in PFA remain unknown, and this signal must be carefully monitored.

Coronary artery spasm has been seen with PFA, particularly when used for currently off-label indications such as the cavotricuspid isthmus line in atrial flutter or the posterolateral mitral isthmus line. This phenomenon, rarely observed with current thermal ablation techniques, relates to the proximity of the electric field to the right coronary artery and the left circumflex artery, respectively. The underlying mechanism of vasospasm is thought to be an injection of current via PFA, as well as a calcium imbalance induced by electroporation. Vasospasm tends to last longer than the stimulus and may persist even after calcium levels normalize [36]. In a small study of 26 patients undergoing mitral isthmus line for AF with either PFA or radiofrequency and with concurrent coronary angiography, coronary spasm was observed in 7 out of 17 (41%) patients undergoing PFA [37]. Most cases were subclinical, and two patients received intracoronary nitroglycerin, with spasm resolution occurring within 5 to 25 minutes. However, the subclinical effects could persist up to 3 months post-procedure based on findings from optical coherence tomography data, including reduced arterial luminal area and increased vascular wall area [26]. At present, high-dose (3 mg) intravenous glyceryl trinitrate has often been administered prophylactically by centers that perform off-label ablation near coronary arteries, with reasonable efficacy, although with an expected decrease in blood pressure [38]. Marked troponin elevation is also seen with PFA, even when applied to the PVs and PW, compared with RF. Likewise, this is related to the number of PFA deliveries [39]. The implication of significant troponin elevation in the absence of symptoms or electrocardiogram (ECG) changes is uncertain.

Intravascular hemolysis is a unique phenomenon associated with PFA. Unintentional electroporation of red blood cells during PFA delivery results in hemoglobin release and depends on the strength of the electric field. Biomarkers of hemolysis, including free hemoglobin, haptoglobin, bilirubin, and lactate dehydrogenase, are affected 24 hours post-procedure [40]. While this has not led to meaningful anemia, it can be associated with hemoglobinuria in more than one-third of cases and subsequent acute kidney impairment. There is a strong correlation between the number of PFA deliveries and the risk of hemolysis. Thus, operators are increasingly wary of the number of applications performed in a single procedure. Administration of intravenous fluids has been adopted to reduce the risk of acute kidney injury, particularly in patients with pre-existing renal impairment [41].

## 6. Procedural Workflow for Pulsed Field Ablation

PFA has significantly improved the procedural workflow and efficiency of AF ablation. Procedural and left atrial catheter dwell time are reduced, which may mitigate the risk of complications and increase procedural volume. Notably, more than 250 PFA procedures are performed annually at our tertiary center. All patients with AF undergoing their first ablation procedure receive PFA. The operator learning curve is relatively quick, with about 20 cases per operator for single-shot technology; however, improved pulmonary vein isolation (PVI) durability is seen after about 60 cases [42]. Despite increased throughput, financial considerations must be considered, given the higher equipment costs currently associated with PFA [43].

The protocol below is a summarized outline of the clinical experience of our center, while recognizing that other centers may have different practices regarding peri-procedural imaging (e.g., transesophageal guidance), anesthesia and ablation techniques, number of PFA applications, anticoagulation, and hospital discharge protocols.

Patients begin fasting from midnight, and therapeutic anticoagulation is typically uninterrupted. Our ablation procedure is performed under general anesthesia, given the availability of anesthetic support, patient comfort, and the reduced risk of patient movement that could alter electroanatomic mapping, where available. However, many centers perform PFA under deep sedation with comparable safety, faster procedural and laboratory occupancy times, higher patient satisfaction, and no difference in arrhythmic recurrence rates compared with general anesthesia [44, 45, 46, 47].

A cardiac anesthetist routinely performs a transesophageal echocardiogram (TOE) to evaluate the left atrial appendage thrombus, guide transseptal puncture, assess PV anatomy, provide a focused assessment of left atrial size and left ventricular function, and assess for pericardial effusion. We do not routinely perform preprocedural computed tomography (CT); instead, anatomical assessment is guided by TOE.

Two femoral venous access sites are obtained under ultrasound guidance, including one for a coronary sinus decapolar diagnostic catheter for backup pacing and to assist with mapping. Intravenous heparin is administered after femoral access, aiming for an activated clotting time (ACT) of 350 seconds; 10,000 to 15,000 units of heparin is administered upfront to achieve the target ACT before left atrial access is rapidly obtained. Trans-septal puncture is performed under fluoroscopic and TOE guidance with either a standard trans-septal sheath with NRG™ RF needle and over-the-wire exchange or with VersaCross Connect™ (Boston Scientific) when using the Farapulse™ catheter, which eliminates the need for sheath exchange. Atropine is administered due to the potential for a vasovagal response following PFA delivery; we have not routinely observed systemic anticholinergic side effects, such as urinary retention. However, anticholinergic side effects have been reported in prospective observational studies, and glycopyrrolate may be more efficacious (fewer vagal responses, asystole, and the need for temporary pacing) and safer (fewer drug-related events) than atropine in this context [48].

**Fig. 1.**
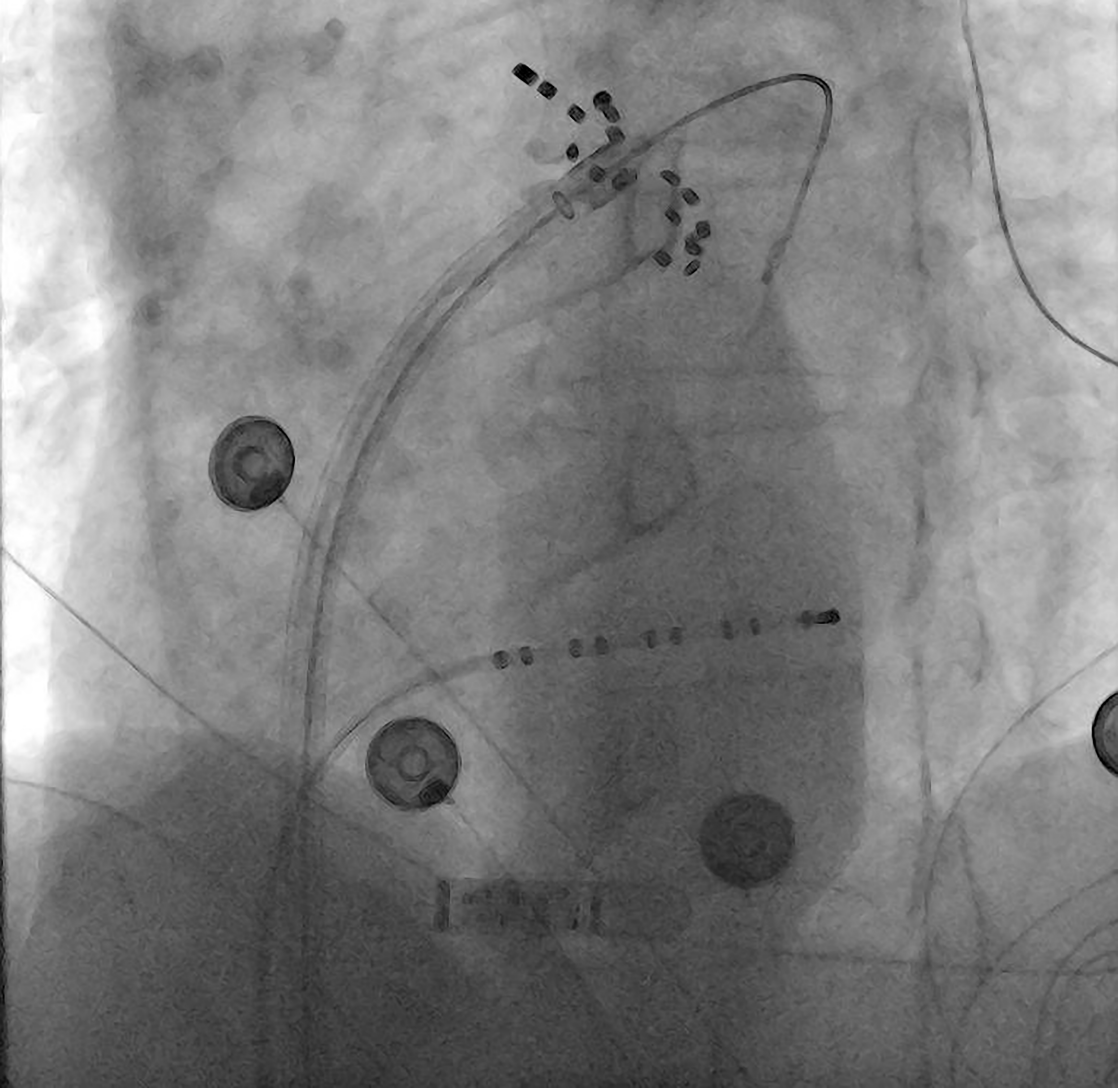
**Pulsed field ablation with Farapulse™**. Farapulse™ catheter using the Faradrive™ (Boston Scientific) 13 French deflectable sheath with an over-the-wire technique to apply PGA to the left pulmonary vein. An implantable loop recorder is also applied *in situ*.

**Fig. 2.**
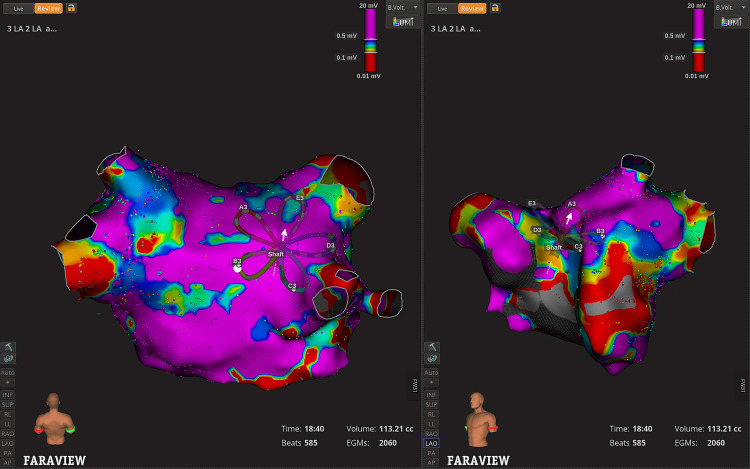
**Pulsed field ablation with Faraview™ electroanatomic mapping**. Bipolar voltage map of the left atrium and pulmonary veins in posterior-anterior (PA) (left side) and Left anterior oblique (LAO) view (middle panel) with local electrogram signals displayed on the right-side panel. The colors represent varying levels of local bipolar voltages: purple indicates areas of high bipolar voltage signals, while red indicates areas of low bipolar voltage signals, correlating with prior ablation.

The subsequent procedural workflow is dependent on the PFA system used. Where concurrent electroanatomic mapping is available, the left atrium and PV anatomy can be mapped before ablation, or ablation can be performed after each PV is mapped. We typically commence ablation with the left-sided PVs, given our catheter is usually already on that side immediately after trans-septal puncture (Fig. 1). A post-ablation map is variably performed to assess PV isolation and evaluate gaps (Fig. 2). After catheter withdrawal from the left atrium, the femoral vein is closed with either a figure-of-eight suture or a percutaneous suture closure device such as Perclose ProStyle™ (Abbott Vascular, USA). A recent randomized trial demonstrated shorter time to ambulation and fewer minor vascular access complications with a percutaneous suture closure device compared with standard figure-of-eight suture [49]. However, routine use of a closure device is limited by financial constraints. Patients remain in bed in a horizontal position for 2 to 4 hours, depending on the groin closure technique. Our center routinely discharges patients the same day unless there is a procedural complication.

## 7. Future Direction

While the published literature regarding PFA for AF is rapidly expanding, many clinical questions remain unanswered. Thus, future research priorities should include (1) evolving catheter design, (2) ongoing monitoring for rare and late complications, (3) lesion optimization and appropriate ablation strategy, particularly for persistent AF, and (4) safety and efficacy of PFA in specific patient groups (Table 1).

### 7.1 Catheter Design

Evolving catheter design is an active process driven by industry, in collaboration with proceduralists. High-density electroanatomic mapping will likely become part of standard PFA systems to guide anatomic delineation, evaluate local bipolar voltage signals, and assist with catheter orientation [50]. There is a growing recognition of contact-sensing information to guide tissue apposition, lesion formation, and depth. Such information may also reduce the risk of inadvertent hemolysis [51]. Preclinical studies highlight the importance of tissue contact for achieving deeper lesions, although the specific role of force-sensing beyond tissue contact remains unclear and warrants further clinical investigation [52, 53, 54]. Studies are also currently underway on smaller-footprint focal PFA catheters as an alternative to conventional RF [55]. A potential role of focal PFA is the improved depth penetration in anatomically challenging areas such as ridges, trabeculated tissue, and the right atrium. There is also likely a role for comparative evaluation between different PFA systems, preferably in a randomized design. Presently, limited comparisons are available from retrospective observational studies. Differences in procedural workflow are recognized and partly influenced by the operator learning curve, with varying extent of low-voltage area and myocardial injury depending on the system [56, 57, 58].

### 7.2 Monitoring for Complications

Although PFA is generally more tissue-selective, the previously described complications still warrant further investigation. The risk of stroke and SCE requires judicious monitoring for neurocognitive function, particularly in the longer term. Coronary spasm may limit the application of PFA in certain areas unless safety can be assured with specific PFA delivery, while maintaining efficacy in the lesion set. Indeed, the longer-term sequelae of acute coronary artery spasm and markedly elevated troponin levels from PFA currently remain unknown. Similarly, optimizing procedural strategies and catheter design to reduce hemolysis and acute kidney injury will be important areas of research to improve the safety of PFA.

### 7.3 Lesion Durability and Appropriate Ablation Strategy 

Lesion durability requires further investigation in the long term. PV reconnections have ranged from 24% to 64% in patients undergoing clinically indicated repeat procedures after initial PFA with a pentaspline catheter [13, 29, 59, 60, 61, 62]. Meanwhile, intracardiac echocardiography can guide catheter contact and reduce reconnection rates [63].

The appropriate ablation strategy, particularly for persistent AF, will need further evaluation in the era of PFA. The CAPLA trial found no difference in freedom from recurrent atrial arrhythmias at 12 months between PV isolation with and without PW isolation in 338 patients with persistent AF using RF ablation [64]. However, the PW was reconnected in 75% of the patients who underwent redo ablation [65]. Caution has historically been exercised when undertaking PW isolation due to its proximity to the esophagus. Thus, greater tissue selectivity with PFA may result in more comprehensive PW isolation with less operator anxiety regarding damage to extracardiac structures. Conversely, the long-term effects of extensive PW isolation with PFA on left atrial function remain uncertain. Only a transient reduction in left atrial function has been observed in early data from 32 patients who had echocardiographic assessment of left atrial strain before PFA, and 1 day and 3 months later; further studies are needed to confirm these findings [66]. The role of PFA for other non-PV triggers of AF, such as the left atrial appendage, ligament of Marshall, coronary sinus, and superior vena cava, will also need to be ascertained. Similarly, the efficacy (lesion durability) and safety (coronary artery spasm) remain uncertain for PFA in cavotricuspid isthmus and mitral isthmus ablation [67, 68, 69].

### 7.4 Threshold to Ablate Specific Patient Groups 

The safety, efficacy, and accessibility of PFA will likely lower the threshold for whom AF ablation is offered based on both patient selection and optimized procedural workflow and safety. Randomized trials have generally supported the notion that upfront AF ablation is associated with reduced symptomatic atrial arrhythmia recurrence and fewer hospitalizations compared with AADs [70, 71]. Patients may elect for early AF ablation rather than medical therapy in the first instance; nonetheless, randomized trial data are needed to evaluate this approach. Similarly, AF ablation may be offered to both younger and older patients at a lower threshold, given a generally more favorable safety profile. Indeed, randomized trials are needed, particularly among older patients, who have been underrepresented in AF ablation trials to date. Notably, PFA has already been demonstrated to be safe in patients with cardiac implantable electronic devices, with no damage to electrical components, in an *ex vivo* study [72]. Only electrical noise-induced brief ventricular pacing inhibition has been observed in a real-world setting across four different PFA systems [73].

## 8. Conclusion

PFA represents a new horizon in the treatment of AF. Procedural efficiency improves, and efficacy is comparable to that of conventional thermal ablation. Early safety data appear acceptable, although specific, uncommon complications unique to PFA require further attention. Given this novel technology, the threshold for offering patients an AF ablation has changed, which has important implications for tackling the burden of AF in our community and improving clinical outcomes for our patients.
